# Personalizing decision-making for persons with Parkinson’s disease: where do we stand and what to improve?

**DOI:** 10.1007/s00415-022-10969-4

**Published:** 2022-01-27

**Authors:** Lieneke van den Heuvel, Marjan J. Meinders, Bart Post, Bastiaan R. Bloem, Anne M. Stiggelbout

**Affiliations:** 1 Donders Institute for Brain, Cognition and Behaviour Department of Neurology Centre of Expertise for Parkinson & Movement Disorders, Radboud University Medical Centre, Nijmegen, The Netherlands; 2 Scientific Centre for Quality of Healthcare, Centre of Expertise for Parkinson & Movement Disorders, Radboud University Medical Centre, Radboud Institute for Health Sciences, Nijmegen, The Netherlands; 3 Medical Decision Making, Department of Biomedical Data Sciences, Leiden University Medical Centre, Leiden, The Netherlands

**Keywords:** Clinical decision-making, Parkinson’s disease, Personalized decision-making, Personalized medicine

## Abstract

**Background:**

The large variety in symptoms and treatment effects across different persons with Parkinson’s disease (PD) warrants a personalized approach, ensuring that the best decision is made for each individual. We aimed to further clarify this process of personalized decision-making, from the perspective of medical professionals.

**Methods:**

We audio-taped 52 consultations with PD patients and their neurologist or PD nurse-specialist, in 6 outpatient clinics. We focused coding of the transcripts on which decisions were made and on if and how decisions were personalized. We subsequently interviewed professionals to elaborate on how and why decisions were personalized, and which decisions would benefit most from a more personalized approach.

**Results:**

Most decisions were related to medication, referral or lifestyle. Professionals balanced clinical factors, including individual (disease-) characteristics, and non-clinical factors, including patients’ preference, for each type of decision. These factors were often not explicitly discussed with the patient. Professionals experienced difficulties in personalizing decisions, mostly because evidence on the impact of characteristics of an individual patient on the outcome of the decision is unavailable. Categories of decisions for which professionals emphasized the importance of a more personalized perspective include choices not only for medication and advanced treatments, but also for referrals, lifestyle and diagnosis.

**Conclusions:**

Clinical decision-making is a complex process, influenced by many different factors that differ for each decision and for each individual. In daily practice, it proves difficult to tailor decisions to individual (disease-) characteristics, probably because sufficient evidence on the impact of these individual characteristics on outcomes is lacking.

## Introduction

Parkinson’s disease (PD) is a chronic, multifaceted disease, with a highly variable presentation and disease course, and with a treatment response that can vary considerably across different individuals [1]. Such a heterogeneous disease demands care that is closely tailored to the needs and characteristics of an individual patient, in a process that has been referred to as personalized medicine (when the clinical profile and individual preferences are considered), and also as ‘precision’ medicine (when the person's individual molecular profile is taken into account) [2].

Personalized care is embedded in evidence-based medicine, which combines three distinct information resources: best available scientific evidence, professional expertise, and the personal needs and preferences of the patient [3, 4]. All three sources have clear value, but also important limitations. For example, current scientific evidence, especially from controlled trials, is often based on selected study populations, and results do not necessarily apply to a person’s specific context. This makes it difficult to predict what decisions would do for individual patients, given their individual (disease-) characteristics [5]. Furthermore, it makes it difficult for clinicians to fully inform patients on what different treatment options would mean for them. There is a need for a more personalized perspective in current medical decision-making, i.e., a perspective that represents the full complexity of an individual patient [3].

The current digital era opens up new opportunities to collect large amounts of data of real-life, unselected patient populations and deep phenotyping, i.e., the comprehensive assessment of a condition using multiple clinical, biological, genetic, imaging, and sensor-based tools [6]. Large datasets can be used to develop fine-grained patient profiles that have the potential to predict best individual therapeutic approaches. Although the first steps towards making personalized predictions for an individual PD patient have been taken in research settings, for example, using patient-specific details to predict motor outcome in DBS [7], the actual evidence supporting the potential of such predictions in PD is still scarce. An important challenge is that it is not yet clear to what extent, and how, decisions in PD are personalized right now, and what kind of decisions would benefit most from a personalized approach.

In this study, we aimed to identify which decisions are made in daily practice, and in which way decision-making in PD is currently personalized, i.e. tailored to the individual patient. Also, we aimed to identify decisions that could particularly benefit from a personalized approach. We used a multimethod approach, including an objective perspective on decision-making in PD by analyzing observations of clinical encounters, combined with the perspective of healthcare professionals using interviews.

## Methods

### Study design

We used a multimethod study design. First, we performed an observational study using audio-recordings from clinical encounters in the outpatient clinic. We focused on three research questions: (1) which decisions were made; (2) how decisions were personalized; and (3) whether the expected effect of the decision was discussed. Next, we performed semi-structured interviews with healthcare professionals experienced in treating PD patients (neurologists, PD nurses and certified PD nurse specialists). The aim of the interviews was to elaborate further on how professionals personalize decisions in PD, which barriers and facilitators they see, and which decisions would benefit most from a personalized approach in their perspective.

### Procedure

#### Observational study

Neurologists, PD nurses and certified PD nurse specialists (jointly referred to as professionals) from six hospitals in the Netherlands agreed to participate in the observation study. We included both university medical centers (*Radboudumc, Nijmegen; MUMC* + *, Maastricht),* non-academic training hospitals *(Rijnstate, Arnhem; Canisius Wilhelmina Ziekenhuis, Nijmegen; Sint Antonius Ziekenhuis, Nieuwegein)* and a general hospital *(Pantein, Boxmeer)* to gain heterogeneity*.* The participating professionals received written information on the study, before signing informed consent. At each site, one observation day was scheduled for data collection, on a day that one to three patients with PD were seen by the professional. The only inclusion criteria were that patients had to be diagnosed with PD (and not parkinsonism) and that it was not their first appointment (as the first appointment is often mainly diagnostic). The only inclusion criterion for professionals was that they were experienced in treating PD patients. We did not preselect participants because we aimed to include a diverse population of patients and professionals to get a realistic view on decisions made in daily practice. The scheduled PD patients received written information on the study prior to the consultation, and if they were willing to participate, informed consent was obtained before the start of the consultation. Next, the professionals audiotaped the consultation. Patients participated in the study only once. We continued collecting data until data saturation was reached.

#### Interview study

All professionals who participated in the observational study were invited to participate in the interview study. The interviews were semi-structured, using a standardized interview guide based on the observations, with open-ended questions. LH, MM and AS developed the interview guide (Appendix 1), which was then discussed with members of the research team who were not involved in the original development of the guide (BP and BB). All interviews were performed by LH and were audio-taped for analyzing purposes. They lasted for 30–40 min, and all but one interviews were performed by phone, for practical reasons. We continued collecting data until data saturation was reached.

### Coding and analysis

The audio-recordings from the observational study and the interview study were transcribed verbatim. The transcripts were analyzed using the framework method [8]. This method consists of seven phases: (1) transcription; (2) familiarizing with the data; (3) coding; (4) developing a working analytical framework; (5) applying the analytical framework; (6) charting data into the framework matrix; (7) interpreting the data [8]. We followed the recommendations outlined in the COREQ criteria as much as possible to analyze and report qualitative data [9].

#### Observations

Though consultations have a high information density, we only coded pre-defined topics, based on our three research questions. First, we identified and classified clinical decisions. We defined a clinical decision as follows: “*A verbal statement committing to a particular course of clinically relevant action or deferment of choice that could alter the patient's current or planned management”* [10, 11]. For our study, we extended Braddock's original definition, to include “deferment” as a valid outcome. All decisions should be at least to some extent, related to PD. Second, we coded individual (disease-) characteristics of the patient that could potentially influence the decision. Third, we coded if and how the expected outcome of the decision for that patient was discussed during the consultation. We quantitatively analyzed the number of decisions. Furthermore, we used a qualitative approach to analyze how decisions were personalized. We chose a qualitative approach because cues on personalization were sometimes subtle and a concise definition of what a personalized decision is is lacking.

Coding was performed by two independent researchers, in ATLAS.ti 8.4.20 software. The first coder (LH) had full knowledge of the terminology used by professionals and patients, informed by her professional background as a medical doctor and clinical experience as a neurologist in training. The second coder (CK) had experience in qualitative research methods but no prior experience in working with PD patients. Two transcripts were also coded independently by two other members of the research team (MM and AS). Differences in interpretation were resolved by consensus in group discussions to ensure reliable and transparent coding. Results were discussed with all members of the research team (MM, AS, BP and BB). MM has a background in PD research, AS has full experience in using qualitative research methods for analyzing medical decision-making and some experience with decision-making in PD. BP and BB both have full experience in treating PD patients.

#### Interviews

For the coding of the interview study, we focused on the items relevant for our research question. This included individual factors important when making decisions; barriers and facilitators in personalizing decisions; decisions that should or should not be personalized (for example, by a prediction model) and the reasons why; and preferred outcomes to predict. Coding was performed by two independent researchers (LH and BS) and three interviews were also independently coded by two other members of the research team (MM and AS). BS had some prior experience with qualitative research methods in PD. Differences in interpretation were resolved in group discussions. Results were discussed with all members of the research team.

### Ethical statement

The study protocol was approved by the Medical Ethics Committee of the Radboud university medical center and registered as 2018-4404. The Local Ethics Committees from all participating institutions approved the study. All participants gave written informed consent prior to the audio-recordings and interviews.

## Results

### Demographics

We included 52 audio-recordings from clinical encounters with PD patients from 19 different professionals (13 neurologists, 3 PD nurses and 3 certified PD nurse specialists). We performed 16 interviews with these professionals (11 neurologists, 2 PD nurses and 3 certified PD nurse specialists). Two neurologists and one PD nurse that were included in the observational study were unable to participate in the interview study due to practical reasons, but data saturation had already been reached. See Table 1 for the demographics.

**Table 1:** Demographics of the participants in the observations

Recordings from consultations	
*Professionals*	
Number	19
Work experience (years, mean (SD))	8.7 (7.1)
Gender (*n* (%) men)	8 (42%)
*Patients*	
Number	52
Gender (*n* (%) men)	42 (81%)
Age (years, mean (SD))	67.5 (10.1)
Years since diagnosis (mean (SD))	6.8 (6.0)
H&Y stage (*n*)	
1	9
2	26
3	6
≥ 4	2
Unknown	9
Receiving advanced treatment (*n*)	
Yes	10 (19%)
DBS	8
LCIG	1
CSAI	1
*Consultations*	
Number	52
Consultations per professional (mean (SD))	2.7 (1.1)
Duration (minutes, mean (SD))	31.0 (15.0)
Decisions per consultation (mean (SD))	5.3 (2.2)

*SD* standard deviation, *H&Y* Hoehn and Yahr, *DBS* deep brain stimulation, *LCIG* levodopa carbidopa intestinal gel, *CSAI* continuous subcutaneous apomorphine infusion

### What are we talking about during consultations—what did we observe?

In total, we identified 263 decisions made in 52 consultations (Table 2). Most clinical decisions were related to medication (*n* = 82), referral (*n* = 36) or lifestyle (*n* = 35). Another large group of decisions was logistic decisions, categorized as ‘other’, including the decision to make a follow-up appointment and the decision to issue a prescription. Decisions could either cause a particular course of action or explicitly defer a particular course of action (for example, a decision not to make a referral). Neurologists made relatively more medication decisions, whereas PD nurses and certified PD nurse specialists made relatively more referral and lifestyle decisions.

**Table 2:** Categorization of decisions made during 52 outpatient clinic consultations between professionals and PD patients

Category	Specification
Dopaminergic medication related^†^ (*n* = 57)	Continuation of medication, starting medication, stopping medication, change of dosage, switch to other medication type
Non-dopaminergic medication related^‡^ (*n* = 25)	Continuation of medication, starting medication, stopping medication, change of dosage
Referral related (*n* = 36)	Referral to other healthcare professionals such as allied health care professionals
Lifestyle related (*n* = 35)	Related to e.g., physical activity, structure of the day, naps, diet
Non-medication treatment related (*n* = 16)	Advanced treatment, specific symptomatic treatment
Addition investigation (*n* = 10)	Laboratory tests, electrophysiological tests, cognitive tests, imaging
Other (*n* = 84)	Prescription, follow-up appointment, filling out specific forms

^†^Dopaminergic medication included levodopa, pramipexole, dopamine-agonist not further specified, amantadine, safinamide, selegiline and mucuna pruriens

^‡^Non-dopaminergic medication included macrogel, clonazepam, rivastigmine, CBD oil, propranolol, quetiapine, viagra, codeine, domperidone, flunitrazepam, mirtazapine and not further specified medication to treat hypertension, tremor, nightmares, stomach complaints or bladder dysfunction

### How personalized are decisions in daily practice—what did we observe?

In the observed consultations, both clinical and non-clinical factors were discussed to tailor decisions to the individual patient (Box 1). Clinical factors referred mainly to information derived from the patient’s history and neurological examination, and were phrased generic (i.e., ‘*’based on your condition’’*). Other clinical factors included the effects and side effects of a decision, as experienced by that specific patient in the past. Non-clinical factors included mainly the preference of the patient, but also psychosocial factors and specific circumstances in the patient’s life at that particular moment, or refraining from making a decision because a different approach was chosen first.

We specifically looked for the discussion of an expected outcome of a decision, as this holds important information for a specific patient and this may influence the decision (Box 2). The expected effect of a decision was seldom explicitly discussed, and if it was, professionals often referred to a ‘trial and error approach’, implicitly stating that the effect of the decision is not known yet. In a minority of the cases, the professionals expressed a specific expectation on the outcome of the decision. In these instances, the expectation was often phrased generically and not specified to the individual. When individual expectations were discussed, the discussion focused mainly on medication decisions or decisions related to advanced treatment.

### How do clinicians personalize decisions? The professionals’ view

The interviews showed that there are ‘standard’ decisions and decisions that are more adapted to an individual patient. Standard decisions are made in a comparable manner for all patients, regardless of their individual characteristics. Reasons for a standard decision were that professionals expected the decision to benefit all patients, because there are no alternatives, or because they strictly followed clinical guidelines. Examples of standard decisions mentioned in the interviews included levodopa as first choice treatment, medication startup schedules, referral to allied health professions (e.g. Physiotherapist), lifestyle advice, referral to a PD nurse, the use of melatonin for REM sleep behavior disorder, or advice provided for treating orthostatic hypotension. Some decisions were considered as standard decisions by some professionals in our study, but warrant a more personalized approach according to others.

To tailor a decision to an individual patient, professionals balanced different clinical and non-clinical factors in each decision (Table 3). Several professionals explained that they base their knowledge of the influence of these individual factors, on studies in larger groups. Clinical factors were specifically mentioned as factors that might influence the expected effect or risk of a decision. Non-clinical factors were often mentioned as factors that would predominantly influence the patient’s or professional’s preference. These individual factors interact with each other and clinical factors might have a non-clinical effect (i.e., cognitive dysfunction might also influence the patient’s individual preference) and non-clinical factors might have a clinical effect (i.e., personality can be a risk factor for developing impulse control disorders when using dopamine agonists). Many of the individual factors that professionals balance to tailor a decision were not explicitly discussed with the patient in daily practice, according to the observations.

**Table 3:** Clinical and non-clinical factors identified by professionals as relevant when personalizing decisions in Parkinson’s disease

*Clinical factors*
Motor- and non-motor symptoms, cognition, existing (side-)effects or (side-) effects in the past, disease course, severity of symptoms, comorbidity, biomarkers
*Non-clinical factors*
Patient related: Patient preference, age, gender, personality*, lifestyle, self-sustainability, personal context, the presence of an informal caregiver, work situation, educational level, time planning, expected treatment adherence, decision-making capacity, coping, degree of involvement in one’s own illness; disease insight; self-management of the patient, stress Physician and practice related: Relationship between professionals and patient, intuition professional, personality of the professional, duration of the consultation, opinion from colleagues, involvement of the PD nurse, preference of the professional Decision related: Intervention intensity, number of available options, available evidence, importance of the decision

### Which decisions should be personalized?

We asked professionals on which decisions they would like to have more information about the impact of that decision for an individual patient. We identified five decision categories: medication-related, advanced treatment-related, referral-related, lifestyle-related and diagnosis-related (Table 4). Decisions focus on ‘Which decision is best for this patient?’, ‘When should this decision be made for this patient?’, ‘Which (side) effect(s) can be expected when making this decision for this patient?’. For some decisions, mainly medication and advanced treatment related decisions, professionals specifically mentioned that a prediction model could be useful for answering these questions.

**Table 4:** Decisions that should be more personalized. Decisions regarding medication and advanced treatment were also most often specifically mentioned as decisions in which prediction models could support personalized decision

Decision category that should be more personalized	Explanation
Medication related	Which side effects will this patient develop? (In particular the risk of developing impulse control disorders on dopamine drug agonist therapy) Which medication type is best for this patient? When to start treatment in this patient? When to increase or decrease the dosage?
Advanced treatment related	When to start advanced treatment for this patient? Which advanced treatment is best for this patient? What (side-)effects can this patient expect?
Referral related	What to expect and when to refer this patient (most often mention for allied healthcare professions)
Lifestyle related	What to expect from lifestyle changes in this patient?
Diagnosis related	How certain is the diagnosis in this patient?

There was some overlap with decisions that, according to professionals, would not, or to a lesser extent, benefit from more information about the impact for an individual patient. Medication-related decisions were mentioned most often (in particular when to start treatment and how to increase the dosage), because the lack of options and the lack of a current dilemma*.* Referral-related decisions were also mentioned because there is little room for mistakes*.* Two participants mentioned lifestyle changes, as these have to do more with motivation than with knowledge about the effect, or because these are less relevant to personalize since it would be beneficial for everybody.

### Which outcomes to use in personalization?

We asked professionals what outcomes would be important for them and for their patients to personalize information (Table 5). These outcomes included those directly related to a decision (e.g., if I make this decision now, how will this affect the quality of life for this patient). Furthermore, it included outcomes focused on prognosis, not necessarily related to a decision or intervention. Several professionals explained that certain outcomes would be particularly relevant for themselves and not necessarily for the patient. The most common example was the prognosis of cognitive decline, where professionals indicate that this would help them to tailor decisions to an individual; however, they noted that patients would not necessarily benefit from knowing their prognosis.

**Table 5:** Outcomes on which clinicians prefer to have more personalized information

Outcome category	Specification
Effect of the intervention	Effect in general; on–off time and motor fluctuations
Risk of side effects	Risk of developing ICD or cognitive problems on dopamine-agonist drug therapy; side effects in general
Risk of complications	Risk of complications in general
Motor symptoms	Mobility; falls; motor symptoms in general; swallowing difficulties; tremor
Non-motor symptoms	Non-motor symptoms in general; psychiatric problems; depression
Quality of life	Different aspects of quality of life
Being independent	Independent in mobility; independent in living situation/time to nursing home; independent in daily life; work participation; independent in general; being able to carry out hobbies
Prognosis	Prognosis regarding cognitive decline; prognosis on disease course; life expectancy

### Barriers and facilitators personalizing decisions

The two most important barriers to tailor decisions to individual patients were (1) difficulty to predict the effect of a decision for an individual patient, and (2) the lack of information on patient or disease variables. For the latter, professionals mentioned as the most important hindering factor that they only see patients for short amounts of time in a clinical setting, and therefore do not have sufficient information on their functioning in daily life. Other barriers included a lack of experience of the professional, individual disease course being difficult to predict, lack of information on the effect of individual disease characteristics, side effects being difficult to predict, lack of trials comparing different treatment options (many treatments have each been compared to placebo, but never back to back to other related effective treatments), effects at the group level being difficult to translate to an individual patient.

Facilitators included mainly opposites of the barriers, such as having more knowledge on the effect of an intervention for an individual patient, and knowing the patient better and longer. Two participants said that more self-monitoring by patients would provide better individual patient- and disease information, which would help them in personalizing decisions.

Box 1. Illustrative quotes for factors discussed in the consultations to personalize decisions **Patient history and clinical examination**

*’I do not think I can make you happier with more or different medication, based on your condition at this moment, hours after your last dose. ‘’* Neurologist-002.

**Effect and side effects in the past**

‘’*You have tried amantadine in the past, but that caused you side-effects.’’* Neurologist-021.

*‘’You have used this long-acting medication before, and we were not sure that there was an effect. But I think that we should try it’’* Neurologist-014.

**Patient preference**

*‘’Well, I think that you must experience what you feel best about’’ ‘’It is subjective, you may have a preference for pills above technology, but it is an option.’’* Neurologist-005.

*‘’Are you the type of person for rest?* <  > *Would that help you?’’ ‘’You can indeed see for yourself whether that is something that helps you.’’* Neurologist-011.

**Psychosocial factors and specific circumstances at that moment**

*‘’It is possible that, when your husband is getting better and you are back at home together, the Parkinson gets better and that the long-acting Levodopa is not needed anymore.’’* Neurologist-037.

*‘’The bed remains the same, but maybe in your new house she [occupational therapist] will notice things and she could say ‘I would do this or this’. That can just help you in the future and then you only have to change it once. It can help you.’’* PDnurse-011.

**Refraining from making a decision because a different approach was chosen first**

*‘’Maybe we can increase the dosage, but let’s wait to see how it will go’’* Neurologist-035.

Not specified.

*‘’Yes, a little rigidity of those vocal cords. We see that in Parkinson's disease. And when people, like you, have that problem, we often suggest going to a speech therapist for a while. If you would like that.’’* PDnurse-012.

Box 2. Illustrative quotes for approaches to discuss the expected outcome of a decision. **Trial and error approach**

*‘’Maybe there will be days that it helps you, maybe there will be days that it does not’’* PDnurse-010.

*‘’Let’s see what helps you’’* PDnurse-012.

*‘’You can continue if you experience a good effect, if there is no effect then you can go back to the old dosage’’* Neurologist-016.

**Specific expectation, not known what it was based on.**

*‘’I would not expect miracles from this medication, but it is an option to try it’’* Neurologist-027.

*‘’I think this long-acting Levodopa can help you during the night’’* Neurologist-037.

*‘’What you can notice is that walking becomes a bit smoother, the hands become a little less stiff’’* Neurologist-022.

**Specific expectation, based on individual cues.**

*‘’When I see your symptoms, they are clearly visible, and those should react on medication’’* Neurologist-022.

*‘’I do not expect side effects. The only thing you should pay attention to is a lowering of the blood pressure during the night. However that is something you do not have during daytime with much higher dosage of Levodopa, so I do not expect this to happen.‘’* Neurologist-014.

*‘’You can get as good as you are at your best with pills for slowness and stiffness* <  > *we can achieve that with the operation.’’* Neurologist-007.

## Discussion

The complexity of PD, including the large variety in symptoms and treatment effects across different patients, necessitates a highly personalized approach in which the best decision is made for each individual patient. The chronic and highly heterogeneous nature of PD, including a unique combination of motor and non-motor symptoms, also makes this a potential model condition for other chronic (neurological) diseases, especially diseases in which interventions that account for the heterogeneity of the disease are lacking. In this study, we explored how personalized decision-making is taking place in current clinical practice and what kind of decisions would benefit most from a more personalized approach. We explored this from the perspective of healthcare professionals regularly involved in the management of persons with PD (neurologists, PD nurses and nurse specialists).

The considerable number of medication-related and not medication-related decisions made in daily practice highlights the diverse role of professionals when treating PD patients. Clinical decision-making is a complex process, influenced by many different clinical and non-clinical factors that differ for each decision and for each individual [12].

Professionals in our study mentioned many different factors influencing their decision-making, ranging from ‘hard core’ clinical factors that directly influence the expected (side-) effect of decisions, to ‘softer’ factors that might affect patient preference. A challenge is that individual factors often interact in a complex manner, and single factors might influence decisions in multiple ways. For example, medication adherence is an important factor when choosing treatment regimes, but is influenced by mood disorders, cognition, poor symptom control, poor quality of life, younger age/longer disease duration, regimen complexity, risk taking behaviour, poor knowledge of PD, lack of spouse/partner, low income, maintaining employment and gender [13]. Some decisions, such as referral to physiotherapy or lifestyle changes, are considered as standard decisions by some professionals in our study, while others advocate a more personalized approach for these decisions. This is interesting, given that decisions such as lifestyle modifications ask for changes that need to fit into the personal life of individuals. Discussing the individual expected effect of the intervention, using a motivational interviewing technique, might help patients to initiate and adhere to lifestyle modifications.

It is interesting to see how different clinical and non-clinical factors that influence personalized decision-making find their way into the consultation room, and importantly, how this process can be optimized further and implemented for all the different professional disciplines that are involved in the management of PD. Even though professionals in our study indicated in the interviews that they use many different factors to tailor decisions to individual patients, these factors were often not explicitly discussed in daily practice. Furthermore, we noticed that the expected effect of a decision was seldom discussed, and if so, professionals mainly referred to a ‘trial and error approach’. A more specific expectation of the effect of a decision for an individual patient was described in generic terms without stating why that was expected for that particular patient. This points out that it is still difficult to deliver care that is tailored to an individual patient. It would also be interesting to see whether a fuller discussion of the factors used to tailor the decision, or a discussion of the expected outcomes for the individual, would lead to better treatment adherence. Studying adherence was beyond the scope of this study.

The most likely explanation for not discussing individual decision outcomes in this study is the difficulty to predict the effect of a decision for an individual patient, which was indeed mentioned as the most important barrier for being able to make truly personalized decisions. Sufficient evidence on the impact of individual patient- and disease characteristics on outcomes is lacking, as are trials comparing different treatment options for different types of patients. Efforts are made to develop more personalized predictive algorithms, using large observational datasets with structured and unstructured data from large populations of real life patients [14, 15]. Machine learning techniques, i.e., analytics that learn automatically from data, can be used to find specific patterns and profiles in large datasets to make more personalized predictions [14]. These are promising developments that might help to overcome the barrier of predicting patient outcome on individual profile level. The question remains whether these technological approaches will allow us to build personal disease profiles that are sufficiently fine-grained to make truly individualized decisions. It is perhaps more likely to expect that machine learning and other comparable approaches will allow us to build a set of subgroups, with much more detail than the rather crude subtyping that is currently available, allowing us to make at least decisions tailored to a specific subtype of PD [1]. Such personalized predictions could ideally be integrated in a shared decision-making process where a patient and professional make a joint decision on what is best for that patient at that moment.

Professionals also mentioned a lack of information on patient or disease variables, for example due to short consultations or not having sufficient information on their at-home situation, hindering their capability to personalize decision-making. This is not surprising, because in the current health care system, the presence and severity of symptoms is as of yet mostly assessed through open-ended queries and brief in-clinic observations. This holds a risk of missing important contextual information, and makes it difficult to provide a complete picture of the patient’s functioning in daily life [16]. Increasingly however, patient-reported outcome measures are being implemented, to assist in monitoring the disease over time and to facilitate patient-professional communication [17]. Newer, digital methods, including continuous, objective monitoring in a home-based setting (such as wearable sensors, e-diaries and smartphone applications), should further enable us to capture a wider range of individualized motor, non-motor, and circadian complex fluctuations with greater accuracy [15, 18, 19]. But even though these new methods are promising for gathering more accurate individual information, such methods are still mainly under construction. Even if applied, the application is mainly restricted to research settings, and most wearable sensor approaches are not (yet) integrated into daily clinical care.

To guide future developments in personalized decision-making, we need reliable predictive algorithms on the individual effect of medical treatment, advanced treatment, referrals and lifestyle changes and the accuracy of the individual diagnosis. Currently, initiatives to make personalized predictions focus on advanced treatment (for example, predicting motor outcome in patients with DBS [7]), diagnosis [20], prognosis [21] or on predicting specific symptoms (such as predicting depression in PD [22]). These initiatives are definitely important, but other categories such as referrals and lifestyle decisions should not be overlooked. Outcomes of such personalized predictions should not only include motor symptoms, but they should also cover non-motor symptoms, as patients consistently indicate the importance of non-motor outcomes across different disease stages [23, 24]. For example, the main patient selected outcomes for advanced treatment in a recent review included quality of life, activities of daily living, ON and OFF time, and adverse events [25]. Professionals in our study also preferred more personalized information on prognosis of cognitive decline and life expectancy. The literature shows that a quarter of PD patients prefer to have information on life expectancy early on in the disease [26].

The major strength of this study is that we are the first who evaluated current personalized decision-making in PD in a systematic way. Another strength is that we used a multi-method design to come as close as possible to daily practice. Also, we included a broad group of professionals from different hospitals which makes our results widely applicable. Neurologists and PD nurse specialists make different decisions (i.e. neurologists make more medication-related decisions and PD nurse specialists more referral-related decisions), and by including both we captured a representative spectrum of clinical decisions. This study is not without limitations. First, we analyzed how decisions were personalized using recordings of consultations. Consequently, we missed nonverbal communication, which is a crucial part of communication during a consultation. However, we focused this study on elements of personalized decision-making that were explicitly discussed during the consultations, and we do not think that including analysis of nonverbal communication would significantly affect our results. Second, we looked at the perspective of the professional. To get a complete view on current gaps in personalized decision-making, and to prioritize which challenges in personalized decision-making should be addressed first, it is indispensable to include the perspective of the patient as well. Also, when personalized predictive algorithms are developed to improve personalized care, such models need to be designed around the preferences of the people they aim to serve. This means that the perspective of persons with PD on the use of personalized predictive models in clinical practice needs to be evaluated. Third, while this is mainly a qualitative study, where we focused on how decisions are personalized, it was not possible to quantitatively express the extent of personalized decision-making and compare this between groups. Fourth, this study was relatively small and only performed in The Netherlands. Professionals from different countries might differ in how they personalize decisions in daily practice, for example, due to differences in medical training, or even due to cultural differences.

In conclusion, this study showed that current decision-making is still a long way from being truly personalized. Sufficient evidence about the impact of individual (disease-) characteristics on outcomes that matter most to patients is lacking. Personalized predictive algorithms, predicting the effects of clinical decisions for individual patients, could ideally be integrated in a shared decision-making process where a patient and professional make a joint decision on what is best for that patient at that moment.

## Appendix 1

**Interview guide** (original in Dutch).

How are decisions being personalized now?

1. How personalized do you think decisions can be made right now? Does it matter what kind of decision is made? Can you give examples? How do you personalize decisions?
2. Are there decisions where you find it difficult to provide the patient with good personalized advice? What kind of decisions are these and what makes it difficult to personalize this advice? How do you do this now? What would it take to make this easier?
3. If you want to make a decision as personalized as possible, which factors do you take into account? How far can you go in this, do you, for example, distinguish main groups (such as age and gender) or is it also possible at n = 1 level? Do you make use of scientific information (and is this sufficiently available)? If not, how do you personalize?
4. In the audio recordings we made, we saw different types of decisions, including medication-related decisions, lifestyle decisions and referrals to other specialists or paramedics. Do you think there is a difference between these types in how personalized these decisions can be made?
5. In your view, are there any drawbacks to personalizing decisions based on individual patient characteristics?

Which decisions would benefit from more personalized advice?

6. Suppose everything is possible, in which decisions would you like to give your patient more personalized information or advice? What do you think this information should consist of? On what outcome would you like to provide more personalized information?
7. Suppose it is possible to develop a personalized prediction model that shows exactly the short and long-term effect of a certain decision or advice for that specific patient, what is a decision in which this has added value in your opinion? And why?
